# The Positive Personality Model (PPM): Exploring a New Conceptual Framework for Personality Assessment

**DOI:** 10.3389/fpsyg.2018.02027

**Published:** 2018-10-25

**Authors:** Guadalupe de la Iglesia, Alejandro Castro Solano

**Affiliations:** ^1^Consejo Nacional de Investigaciones Científicas y Técnicas, Buenos Aires, Argentina; ^2^Departamento de Psicología, Facultad de Ciencias Sociales, Universidad de Palermo, Buenos Aires, Argentina; ^3^Facultad de Psicología, Universidad de Buenos Aires, Buenos Aires, Argentina

**Keywords:** positive personality, personality, assessment, positive traits, DSM-5, PPM

## Abstract

The aim of this paper is to explore a new framework for personality assessment that may function as sanity nosology of personality traits: the Positive Personality Model (PPM). The recent publication of DSM-5 created the opportunity to assess personality traits as dimensional constructs (American Psychiatric Association, 2013). In Section III, five maladaptive personality traits are proposed as the maladaptive versions of Five Factor Model (FFM) traits (Costa and McCrae, 1985). This approach draws on the existing idea of conceptualizing pathological and typical personality traits as part of a *continuum*. It places DSM-5′s maladaptive traits in a sickness pole and FFM’s traits in a “typical” pole. This spectrum, however, does not include a positive perspective that represents healthy behavior: a sanity nosology. The Positive Traits Inventory-5 (PTI*-*5; de la Iglesia and Castro Solano, 2018) is a measure designed to assess the positive reverse of the Personality Inventory for DSM-5-Adult (PID-5; Krueger et al., 2013). The 220 positive personality criteria were studied psychometrically using a sample of 1902 Argentinean adults from the general population (*M*_age_ = 39.10, *SD* = 13.81, Min = 18, and Max = 83; 50.1% females, 49.9% males). Exploratory and confirmatory factor analyses resulted in a five-factor solution. The dimensions were labeled Sprightliness, Integrity, Serenity, Moderation, and Humanity and subsumed under the denomination of PPM. Analyses of convergent validity provided some grounds for interpreting the five positive traits as positive versions of the pathological traits and the typical traits. When tested for its predictive capability on mental health, the PPM outperformed the variance explained by the FFM. It is concluded that the PPM may constitute a positive pole in the continuum of personality traits –possibly functioning as a sanity nosology– and that it is somewhat more related to optimal functioning than typical trait models. The PPM should be confirmed in other populations, its predictive capability ought to be tested with other relevant variables, and longitudinal studies should be done to analyze the stability of the traits over time.

## Introduction

During most of the 20th century, psychology has made significant progress in diagnosing and treating psychological disorders. As Millon (1996) stated, the medical model that specifies that mental health is the absence of pathology had set the course of action in the psychological arena for a long time. However, nowadays, it is known that the lack of psychopathological symptoms does not guarantee an optimal life functioning or a good quality of life (Seligman and Csikszentmihalyi, 2014; World Health Organization [WHO], 2014).

The debate regarding psychopathology nosologies is centered on outlining which traits are not desirable in people. However, when trying to describe and diagnose healthy behavior, the approach is not so clear. Although some models have been proposed, such as Peterson and Seligman’s (2004) classification of character strengths and virtues known as the *Values in Action* (VIA) model, scientists have not yet agreed upon a commonly used manual that comprises healthy symptoms. In other words, there is not yet a clear and agreed idea of how healthy people should be like or behave. Therefore, there is not yet a sanity nosology that comprises the elements for assessing the presence of mental health (Wakefield, 1992; Sadler and Fulford, 2006; Leising, 2008; Leising et al., 2009).

Over the years, the notion that normality and pathology should be organized in a unique conceptual framework has received greater acknowledgment (e.g., Millon, 2011). However, to date, there is no integrated classification of psychopathology and normality that is widely accepted by theorists and clinicians. One of Peterson’s latest works attempted to develop a new mental illness nosology starting from the VIA sanity nosology and stating the pathological versions of each of its aspects (Seligman, 2014). Although remarkably interesting, this work remains unfinished.

In the field of *personality* assessment, Section III of the latest edition of DSM (American Psychiatric Association, 2013) presents a dimensional approach for assessing personality disorders (PDs) (Hopwood et al., 2013). In this model, personality traits are conceived as consistent patterns of behavior, emotion, and thought (e.g., Allport, 1937; Cattell, 1965) of a dimensional nature (Goldberg, 1993). The dimensional approach– as opposed to the classical categorical approach– is thought to be closer to reality and to have greater empirical support since it is hypothesized to more adequately represent PDs’ degrees of severity and comorbidity among different diagnoses (Clark et al., 1997; Cloninger, 2000; Trull and Durrett, 2005; Widiger and Samuel, 2005).

Krueger et al. (2013) designed the *Personality Inventory for DSM-5* (PID-5) to measure the five PDs’ maladaptive traits of Section III from an empirical and dimensional perspective. This instrument assesses five personality dimensions: Negative Affect, Detachment, Antagonism, Disinhibition, and Psychoticism. It is considered to be the pathological version of the FFM personality traits (Costa and McCrae, 1985), a historically and emphatically rejected model for diagnosing PD (Millon, 1996; Krueger et al., 2013). Although these personality traits are presented in the DSM-5 as the opposite poles of the FFM traits, no further description is specified (American Psychiatric Association, 2013).

The idea of conceptualizing pathological and typical personality as part of a *continuum* is not new. It states that the dividing line between health and sickness is relative and thus, pathology and normality ought to be seen as arbitrary points of such a continuum (Strack and Lorr, 1994). Clear precedents of this concept can be found in the works of Leary (1957) and Gunderson (1979), who stated that normal and abnormal behaviors belong to a unique continuum of personality, where differences rely on quantities, not qualities. Millon (2011, 2012) emphasized this notion in terms of adaptation and asserted that adaptive and maladaptive functioning should be conceptualized as points of a unique phenomenon. For example, in an attempt to integrate typical and pathological aspects of personality in a solo continuum, Millon (2011) proposed the existence of 15 personality *spectrums.*

A proposal centered on stating the reverse and healthy version of the pathological signs included in the current DSM should lead to a conceptual framework that comprises psychopathology and sanity. In this sense, Millon (2003) stated that there is no debate regarding the indirect relationship between health and disease, but that it does not suffice to describe healthy personality as the absence of disorder. He adds that “positive personality functioning must comprise elements beyond mere non-normality or abnormality” (p. 11). Designing a manual of sanity for personality traits would necessarily entail the use of a *value system* and the introduction of a *value dimension of personality* (Schwartz, 1992; Leising et al., 2009). Originally, personality traits had a neutral connotation to merely describe how people are like and to detect individual differences (Nicholson, 1998). Current taxonomies of personality traits, although useful, do not include a value dimension. It was not until recently that psychologists took an interest in recovering concepts aligned with positive psychology traits (e.g., Seligman et al., 2005; Park and Peterson, 2009; Morales-Vives et al., 2014). Two main empirical approaches may be identified in an attempt to study and categorize *human positive characteristics* (Chow, 2002): (a) those guided by data (*data-driven*), and (b) those guided by theories (*theory-driven*).

Data-driven models derived from nosologies developed within a psycholexical approach. This perspective consists of a series of inductive studies in which groups of elements –positive characteristics or traits– are identified in order to find classifications that may be generalized to several populations. This perspective considers that individual differences in positive traits are coded in people’s natural language, especially in implicit expectation/ideas of how a highly valued individual is like or behaves. Some examples of this approach are the work of Walker and Pitts (1998); Cawley et al. (2000); De Raad and Barelds (2008); De Raad et al. (2010); and Morales-Vives et al. (2014). In Argentina, Cosentino and Castro Solano (2017) used the lexical approach and identified human positive psychological characteristics from laypeople’s point of view. A total of 745 individuals were asked to think of a person they admired, not in physical or economic terms, and to enumerate up to seven words that described the characteristics they admired the most. A list of 854 socially shared human positive psychological characteristics was obtained, and after a series of analyses, those words were grouped in five positive factors. Consequently, the High Five Model (HFM) was postulated, comprising the following dimensions: erudition, peace, cheerfulness, honesty, and tenacity. When compared to the FFM, the HFM was a better predictor of positive mental health.

On the other hand, theory-driven approaches are theoretical-rational approximations initiated in a particular theory and then empirically confirmed. The design of the VIA model (Peterson and Seligman, 2004) is a clear example. This classification of six virtues and 24 strengths was guided by expert consensus and has an extended use (e.g., Castro Solano, 2014; Cosentino, 2014; Castro Solano and Cosentino, 2016). Additionally, it has great empirical support regarding the prediction of psychological outcomes, such as increases in happiness and decreases in depressive symptoms (Proyer et al., 2015), and increments in coping with work stress (Harzer and Ruch, 2015), in work productivity (Lavy and Littman-Ovadia, 2017), in well-being (Martínez Martí and Ruch, 2014) and in academic achievement (Wagner and Ruch, 2015), among many others (VIA Institute on Character, 2017). As with many psychological constructs, there is not much evidence of factorial structure and transcultural validity of the VIA model (e.g., Peterson and Seligman, 2004; Otake et al., 2005; Brdar and Kashdan, 2010; Ruch et al., 2010; Shryack et al., 2010; Singh and Choubisa, 2010; Cosentino, 2011; Duan et al., 2012; McGrath, 2014; Ng et al., 2017). For instance, Ruch and Proyer (2015) concluded that the VIA model should be adjusted to allow strengths to be subsumed under more than one virtue, or that some of the strength definitions should be modified in order to represent only one virtue. Consequently, they suggested that the most appropriate approach to conceptually test the VIA model is by expert judgment rather than by factor analysis. In a similar line, Najderska and Cieciuch (2018) used a person-centered approach to study the structure of character strengths and concluded that a factorization of the VIA model may be described by using the FFM metatraits.

Also within the theory-driven approach, Leising et al. (2009) developed a model by formulating the inverted criteria for each of the PD criteria included in DSM-IV-TR. From this analysis, 10 clusters of aspects implicitly valued as healthy in the DSM were identified. Firstly, a series of statements that expressed the semantic opposite of the 79 PD criteria of DSM-IV-TR were formulated. However, as removing the negative formulation was not enough in some cases, some positive statements were developed. Then, a total of 28 judges grouped the statements by similarity. Also, a hierarchical analysis of the statements suggested a 10-cluster solution, which included 27 subclusters, as the most appropriate model. The 10 clusters were: (1) be self-reliant and independent; (2) be self-confident, but in a realistic manner; (3) get along with others; (4) tolerate uncertainty and imperfection; (5) look for the good in people; (6) be conventional; (7) have self-control; (8) connect with others emotionally and treat them fairly; (9) enjoy social relationships and activities; and (10) be trusting.

Additionally, from a theory-driven approach, Huppert and So (2013) developed a measurement of flourishing –optimal functioning and well-being–, using items from the European Social Survey. Firstly, the opposite criteria for generalized anxiety disorder and major depressive disorder as described in DSM-IV-TR (American Psychiatric Association, 2002) were formulated by listing all the symptoms and enunciating them in a positive wording. In other words, the statements not only refer to the absence of the symptoms –their neutral version– but also highlight the positive aspect beyond the neutral point. Afterward, the new statements were grouped in positive features that combined feelings and functioning.

Taking into account the sickness-health continuum and the latest pathology nosology for PDs, de la Iglesia and Castro Solano (2018) proposed the Positive Personality Model (PPM). This model includes five dimensions that aim to represent the positive opposites of the dimensional classification of PD introduced in the DSM-5 (American Psychiatric Association, 2013), and it was based particularly on the pathological indicators included in PID-5 (Krueger et al., 2013). The procedure involved formulating the positive version of each of the 220 PID-5 statements and grouping those items by domains and facets as stipulated in PID-5 scoring instructions, conjointly scrutinizing all of its elements –items and definitions–. Items like “I am a very nervous person,” “It is easy for me to take advantage of others,” or “I am impulsive,” were reformulated in their positive versions as “I am a very calm person,” “It is natural for me to be generous to people,” “I am the type of person that thinks before doing something,” respectively.

The five positive domains of the PPM were named well-being, positive bonds, humanity, moderation, and lucidity. The formulation of the positive versions was somewhat difficult for the Psychoticism domain. For example, the opposite version of “I’ve had experiences that are hard to explain” was “I perceive things as most people do.” Although this statement was a clear opposite, the item seemed strange and lacking a positive element. Some difficulties were also found in the Detachment domain, as its opposite version turned out to be similar to the FFM’s Extraversion dimension. Afterward, a pilot study was conducted and expert judgment was sought to adjust language and expressions of the PPM. This procedure was helpful in providing items that more accurately represent the construct to be assessed, while being more comprehensible to the general population. For example, “I enjoy what happens to me” was reformulated to “I enjoy it when nice things happen to me.” This first version was also studied by means of other preliminary psychometric analyses: internal consistency analysis and convergent validity studies using the PID-5 and the BFI (John et al., 1991). All facets and domains showed good internal consistency (Cronbach’s alphas >0.70), except for the Determination facet, which showed low internal consistency (0.37). Except for the correlations of Lucidity, which were weak, correlations among the PPM domains and the pathological and typical trait measures were as expected: moderate and negative with PID-5′s domains, and moderate and positive with BFI’s factors.

Besides the evidence found to support the PPM, some doubts still remain, particularly regarding the items that make up the Lucidity and Positive Bonds domains as well as the weak correlations between Lucidity and the other measures explored. Additionally, many items generated as the opposite versions of the items of the PID’s secondary facets, which are excluded from the domains scores, seemed extremely important if the aim was to study positive traits –e.g., Humility (opposite of the secondary facet Attention seeking), and Compassion (opposite of the secondary facet Callousness), among others–. Therefore, an exploratory analysis that includes all 220 items and seeks a parsimonious factor structure seems to be the logical next step. Providing additional evidence by studying the 220 positive items could result in the postulation of a positive trait model adjusted to the most current nosology of PDs (DSM-5) and, therefore, a theoretical-rational basis as well. A viable way –though neither the only one nor the best– for establishing a sanity nosology may rely on empirically establishing the “reverse” version of those characteristics that experts have labeled as maladaptive personality traits (Task Force del DSM-5) and placing them on the positive extreme of the sickness-health continuum (Huppert and So, 2013).

Consequently, the objectives of this research were (1) to identify the underlying factorial structure of the 220 positive traits statements; (2) to confirm the isolated structure in a sample other than the one used in the exploratory study; (3) to obtain evidence of convergent validity with instruments of maladaptive personality traits (PID-5) and typical personality traits (BFI); and (4) to determine whether the PPM adds up to the prediction of mental health beyond the variance explained by the typical personality traits –incremental validity–.

The hypotheses formulated were (1) a five-factor structure will be found; (2) the positive traits will correlate negatively and moderately with PID’s maladaptive personality traits and positively but weakly with BFI’s typical personality traits; and (3) the positive traits will have more predictive power over the typical traits when predicting mental health.

## Materials and Methods

### Design and Procedure

The design of this research was cross-sectional. A convenience sample with volunteer participants was obtained in Buenos Aires City in 2016. Advanced psychology students, supervised by a senior researcher, collected data. Participants met the following inclusion criteria: being Argentinean and older than 18 years of age. Adults who were under psychiatric treatment were not included in the assessment. The main sample was obtained by snowball sampling at two different times throughout a year. The first wave of data (subsample A) was used to identify a factor structure for the PTI-5 by exploratory factor analysis (EFAs). The second wave of data (subsample B) was used to run a confirmatory factor analysis (CFA) on the structure found in subsample A. All data collected was used in all other analyses.

### Ethics Statement

Participation was voluntary and anonymous. Participants were informed about the objective of the research and the possibility to refuse or to interrupt their participation at any time. They were asked to give their informed and written consent. No incentives were given either to participants or to data collectors. This research followed the ethical guidelines and was approved by the National Council for Scientific and Technical Research (CONICET), the University of Buenos Aires, and the University of Palermo. A formal evaluation by an ethics committee was not needed due to the research characteristics (voluntary and anonymous participation of adult individuals with informed consent in studies without intervention) as per the mentioned institutions’ guidelines and national regulations. However, the research met the ethics guidelines, and it was tacitly approved by an in-house ethics committee at the Department of Psychology, Universidad de Palermo. The committee is familiarized with all ongoing research and intervenes if necessary.

### Participants

The main sample consisted of 1902 Argentinean adults from the general population (50.1% females, 49.9% males). Mean age was 39.10 years old (*SD* = 13.81, *Min* = 18, and *Max* = 83). Most participants resided in Buenos Aires Autonomous City (61.5%), 25.8% lived in the Greater Buenos Aires, and 12.7% lived in different provinces of the country. Regarding their marital status, 28.8% were married, 26.1% were single, 17% were dating someone, 18.6% were living with a partner, 6.5% were divorced, and 2.9% were widowed. Most of the sample had obtained a high school diploma (48.9%), 38.4% had completed undergraduate studies, 7.1% had finished elementary studies, 4.9 % had finished graduate studies, and a small percentage had not completed elementary studies (0.7%). Regarding their occupation, most participants had paid jobs (83.4%). In terms of socioeconomic status (SES), 67.8% reported middle SES, 19.9% upper-middle SES, 10.7% lower-middle SES, 0.9% high SES, and 0.7% low SES.

Subsample A was composed of 860 participants (49.8% females, 50.2% males) with a mean age of 38.50 (*SD* = 13.46) and subsample B was composed of 1042 participants (50.3% females, 49.7% males) with a mean age of 39.59 (*SD* = 14.08).

### Materials

#### Inventario de Rasgos Positivos-5 –Positive Traits Inventory-5– (PTI-5; de la Iglesia and Castro Solano, 2018)

This 220-items instrument assesses positive traits, as described in the Introduction.

#### The Personality Inventory for DSM-5–Brief Form Adult (PID-5-BF; Krueger et al., 2013)

This instrument is the short version of the 220-item inventory and it assesses the main five domains with 25 items: Antagonism (e.g., “It is not so bad to hurt someone’s feelings,”) Detachment (e.g., “I stay away from romantic relationships,”) Disinhibition (e.g., “I feel as if I acted completely on impulse,”) Negative Affect (e.g., “I worry about almost everything,”) and Psychoticism (e.g., “I have seen things that weren’t really there”). Regarding its psychometric properties, the Argentinean local study provided evidence for the five-factor structure, and of divergent validity and good internal consistency (Góngora and Castro Solano, 2017). In this sample, Cronbach’s alphas for all traits indicated good internal consistency (>0.70).

#### Big Five Inventory (BFI; John et al., 1991)

This instrument measures the five-factor model of personality traits: Extraversion (e.g., “Likes to talk,”) Agreeableness (e.g., “Is very reliable,”) Conscientiousness (e.g., “Is able to finish a task,”) Neuroticism (e.g., “Is depressive or sad,”) and Openness to experience (e.g., “Is curious about things”). The local version consists of 44 items that are answered on a 5-point Likert scale. The local study informed good psychometric properties in terms of construct and external validity as well as internal consistency (Castro Solano and Casullo, 2001). In this sample, the internal consistency of each subscale was good (Cronbach’s alphas >0.70).

#### Mental Health Continuum–Short Form (MHC–SF; Keyes, 2005)

This instrument assesses mental health and consists of three subscales of well-being: emotional (e.g., “Happy,”) social (e.g., “That people are good,”) and psychological (e.g., “That my life has meaning and purpose”). A total score may also be calculated. The 14 items are answered on a 6-point Likert-type scale. The psychometric adaptation to the Argentinean population included CFA and analyses of external validity and internal consistency. The results indicated proper psychometric functioning of the scale (Lupano et al., 2017). The internal consistency in this sample was good for all MHC’s measures (Cronbach’s alphas >0.69).

### Data Analysis

Subsample A was used to study the dimensionality of the PTI-5 using FACTOR software version 10.03.01 (Lorenzo-Seva and Ferrando, 2013). This software was designed specifically to run EFAs, it provides a wide variety of possible procedures of analysis, and it is open access software. Since the 220 items were responded on a Likert-type ordinal scale, a polychoric correlation matrix was used, and parallel analysis (PA) was based on minimum rank factor analysis (MRFA; Timmerman and Lorenzo-Seva, 2011). Since the factors were expected to correlate, the data were rotated using Promin rotation. To obtain a replicable factor structure that was sound and consistent with the background theory, it was established that the final factor structure should have the following characteristics: (1) items with high factor loadings (>0.40) in a single factor; (2) factors with at least five items; (3) factors with reliabilities higher than 0.80; and (4) appropriate item content in terms of coverage of the construct assessed –content validity–.

Then, the model was confirmed in subsample B. It was tested using EQS 6.2. The estimation method was Maximum Likelihood (ML) and, since variables were treated as ordinal, the polychoric matrix was used. When variables are ordinal, this type of matrix is more appropriate (Muthén and Kaplan, 1985; Freiberg Hoffmann et al., 2013). To value the model fit of the five-factor model, different indexes obtained by the robust method were examined: Chi-square (χ^2^), comparative fit index (CFI), Bollen’s fit index (IFI), the standardized root mean-square residual (SRMR), and root mean square error of approximation (RMSEA).

Later, using the main sample and aiming to assess the PTI-5 convergent validity, Pearson product-moment correlations were calculated among the PTI-5, the BFI and the PID-5 scales. Finally, a hierarchical multiple regression analysis was conducted on each measure of the MHC. In step 1, the BFI scales were included as predictors in the first block, and in step 2, the PTI-5 scales were included as a second block.

## Results

### Evidence of Internal Validity

In order to obtain evidence of internal validity, exploratory, and confirmatory factor analyses were calculated. Using subsample A and the set of rules explained in section 2.5, many items were sequentially removed and the analysis was rerun until a solution that satisfied the aforementioned requirements was obtained. The final solution included 60 items grouped in five factors (see Supplementary File). Total explained variance was 59.45% and KMO statistics indicated that sampling was very adequate. Also, reliability coefficients –Cronbach’s, ordinals and Omegas– informed a good internal consistency of each dimension (>0.80).

After studying the factors’ content, they were labeled as Sprightliness (17 items), Integrity (13 items), Serenity (13 items), Moderation (9 items), and Humanity (8 items). Some examples of items for each factor are: *Sprightliness* (“I enjoy life” and “When I have a goal I focus on accomplishing it”), *Integrity* (“I am sincere about my intentions when I ask for help” and “I have flaws, like everyone else”), *Serenity* (“If something bothers me, I try to solve it politely” and “If I disagree with someone, I prefer talking calmly”), *Moderation* (“I avoid unnecessary risks” and “I think about the consequences my actions may have”), and *Humanity* (“I often get involved in charitable causes” and “I help those who are suffering”). Then, Pearson product-moment correlations were calculated among all dimensions (see Supplementary File). Associations were all statistically significant and positive. Effect sizes were moderate.

**FIGURE 1:**
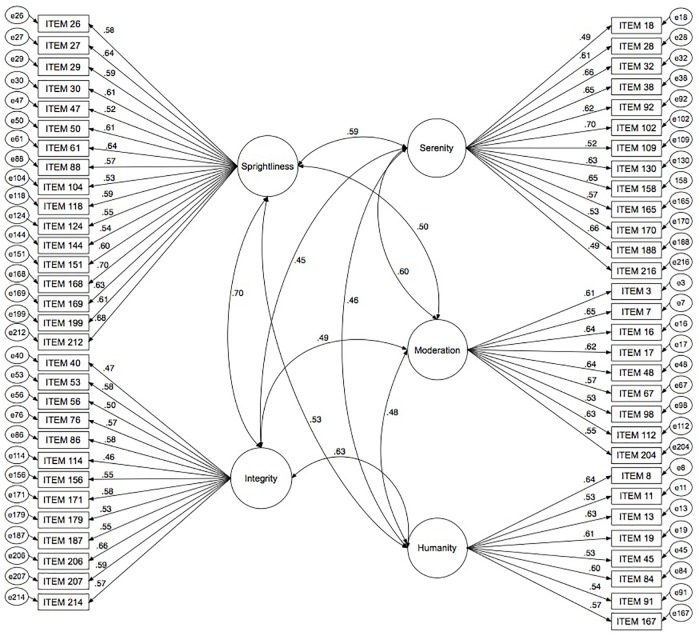
Confirmatory factor analysis of the PTI-5 (*n* = 1042).

For subsample B, a CFA was tested to confirm the factor structure found in subsample A (Figure 1), following the methodology explained. The indexes obtained indicated a good fit: χ^2^ = 9122.88, 1770 *df*, *p* < 0.001, *CFI* = 0.931, *IFI* = 0.931; *SRMR* = 0.062, *RMSEA* = 0.065, 90%CI [0.063,0.066].

### Evidence of Convergent Validity

Evidences of convergent validity (American Educational Research Association, American Psychological Association, and National Council on Measurement in Education, 2014; Coulacoglou and Saklofske, 2017) were studied assessing the correlations between PTI’s positive traits and BFI’s typical traits, and PTI’s positive traits and PID-5′s pathological traits see Table 1. Positive and weak correlations were expected between PTI-5 and BFI’s scales in order to obtain evidence of low convergence between positive and typical traits since they were hypothesized to be related but not equal constructs. The associations obtained were positive and between moderate and weak. Openess to experience, Conscientiousness, and Extraversion were the least related to their positive versions. Neuroticism (reversed) and Agreeableness were moderately related to their positive trait versions. This indicates mixed evidence regarding convergent validity see Table 1. On the other hand, negative and high correlations were expected between the PTI-5 and the PID-5 since positive and pathological traits were hypothesized to be inversely related. The associations found were all negative and medium. Although the associations found were moderate, they were in line with the results expected since they indicate that the positive traits vary in the opposite direction of the pathological traits.

**Table 1:** Correlations among PTI-5, PID-5, and BFI scales (*n* = 1902).

	PTI-5				
	Sprightliness	Integrity	Serenity	Moderation	Humanity
**PID-5**					
Negative affect	−0.316^∗∗^	−0.052^∗^	−0.349^∗∗^	−0.151^∗∗^	0.051^∗^
Detachment	−0.384^∗∗^	−0.244^∗∗^	−0.256^∗∗^	−0.118^∗∗^	−0.267^∗∗^
Antagonism	−0.237^∗∗^	−0.367^∗∗^	−0.290^∗∗^	−0.233^∗∗^	−0.244^∗∗^
Disinhibition	−0.282^∗∗^	−0.201^∗∗^	−0.328^∗∗^	−0.461^∗∗^	−0.117^∗∗^
Psychoticism	−0.336^∗∗^	−0.207^∗∗^	−0.262^∗∗^	−0.306^∗∗^	−0.160^∗∗^
**BFI**					
Neuroticism	−0.404^∗∗^	−0.189^∗∗^	−0.491^∗∗^	−0.130^∗∗^	−0.001
Extraversion	0.368^∗∗^	0.205^∗∗^	0.074^∗∗^	−0.026	0.258^∗∗^
Agreeableness	0.322^∗∗^	0.460^∗∗^	0.438^∗∗^	0.222^∗∗^	0.394^∗∗^
Conscientiousness	0.487^∗∗^	0.343^∗∗^	0.190^∗∗^	0.304^∗∗^	0.186^∗∗^
Openness to experience	0.265^∗∗^	0.228^∗∗^	0.139^∗∗^	−0.008	0.238^∗∗^

*^∗^p < 0.05, ^∗∗^p < 0.01; PTI-5, Positive Trait Inventory for DSM-5; PID-5, Personality Inventory for DSM-5; BFI, Big Five Inventory; n = 1902, except for correlation with PID-5 (n = 1502).*

### Evidence of Incremental Validity

Hierarchical regression analyses were calculated to test if the PTI-5 increased the prediction of well-being beyond the variance explained by personality scales (BFI). Models of steps 1 and 2 (see section “Data Analysis”) were statistically significant in all cases, and the PTI-5 increased the explained variances in all cases (see Table 2).

**Table 2:** Hierarchical multiple regression analyses (*n* = 1902).

		*R*^2^	*F*(*df*)	*p*	*Standardized* β	*p*
**Total Well-being**						
*Step 1 (BFI scales)*		0.222	109.24(5,1896)	<0.001		
	Neuroticism				−0.185	<0.001
	Extraversion				0.222	<0.001
	Agreeableness				0.120	<0.001
	Conscientiousness				0.095	<0.001
	Openness to experience				0.090	<0.001
*Step 2 (BFI and PTI-5 scales)*		0.333	95.87(5,1891)	<0.001		
*p* for *F* change		<0.001				
	Neuroticism				−0.082	0.001
	Extraversion				0.147	<0.001
	Agreeableness				0.106	<0.001
	Conscientiousness				−0.025	0.294
	Openness to experience				0.052	0.012
	Sprightliness				0.410	<0.001
	Integrity				−0.122	<0.001
	Serenity				0.050	0.068
	Moderation				0.011	0.628
	Humanity				0.076	0.002
**Emotional Well-Being**					
*Step 1 (BFI scales)*		0.160	73.38(5,1896)	<0.001		
	Neuroticism				−0.191	<0.001
	Extraversion				0.158	<0.001
	Agreeableness				0.073	0.003
	Conscientiousness				0.113	<0.001
	Openness to experience				0.070	0.002
*Step 2 (BFI and PTI-5 scales)*		0.262	68.62(5,1891)	<0.001		
*p* for *F* change		<0.001				
	Neuroticism				−0.111	0.000
	Extraversion				0.078	0.001
	Agreeableness				0.097	<0.001
	Conscientiousness				−0.035	0.159
	Openness to experience				0.032	0.142
	Sprightliness				0.437	0.000
	Integrity				0.023	0.408
	Serenity				−0.079	0.006
	Moderation				−0.015	0.547
	Humanity				−0.046	0.071
**Social Well-being**					
*Step 1 (BFI scales)*		0.084	35.84(3,1896)	<0.001		
	Neuroticism				−0.132	<0.001
	Extraversion				0.149	<0.001
	Agreeableness				0.127	<0.001
	Conscientiousness				−0.035	0.153
	Openness to experience				0.031	0.196
*Step 2 (BFI and PTI-5 scales)*		0.176	41.47(5,1981)	<0.001		
*p* for *F* change		<0.001				
	Neuroticism				−0.038	0.170
	Extraversion				0.113	<0.001
	Agreeableness				0.112	<0.001
	Conscientiousness				−0.081	0.002
	Openness to experience				0.016	0.484
	Sprightliness				0.239	<0.001
	Integrity				−0.271	<0.001
	Serenity				0.147	<0.001
	Moderation				0.051	0.053
	Humanity				0.140	<0.001
**Psychological Well-being**					
*Step 1 (BFI scales)*		0.242	77.18(5,1896)	<0.001		
	Neuroticism				−0.149	<0.001
	Extraversion				0.225	<0.001
	Agreeableness				0.082	<0.001
	Conscientiousness				0.173	<0.001
	Openness to experience				0.119	<0.001
*Step 2 (BFI and PTI-5 scales)*		0.339	98.29(5,1891)	<0.001		
*p* for *F* change		<0.001				
	Neuroticism				−0.073	0.004
	Extraversion				0.149	<0.001
	Agreeableness				0.056	0.028
	Conscientiousness				0.049	0.038
	Openness to experience				0.076	<0.001
	Sprightliness				0.378	<0.001
	Integrity				0.007	0.777
	Serenity				−0.003	0.924
	Moderation				−0.020	0.402
	Humanity				0.040	0.101

## Discussion

**FIGURE 2:**
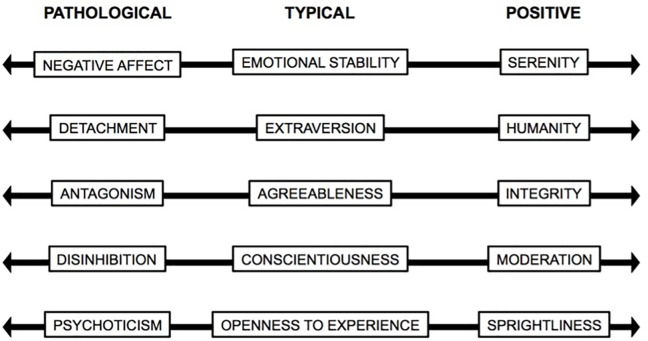
Personality traits continuums.

A classification of positive mental health integrated to the current and most accepted psychopathology nosologies could bring together the study of personality traits by considering the complete sickness-health continuum. Based on this concept, de la Iglesia and Castro Solano (2018) proposed the PPM as the positive version of the maladaptive personality traits model included in section III of DSM-5. Consequently, they generated the positive version of PID-5: the PTI-5. However, to date, psychometric studies conducted on the PTI-5 have been insufficient and have not guaranteed their replicability and generalizability. Firstly, it was necessary to identify the underlying factor structure to the 220 positive statements. The exploratory study presented in this article indicated that a five-factor structure was the most appropriate one. This structure was confirmed in a different sample, contributing evidence of construct validity of the measure. Convergent validity analyses provided mixed evidence regarding the associations with BFI’s and PID-5′s traits. The associations observed provide some grounds to suggest that the five positive trait model may be placed in the positive pole of the continuum generated from the five personality pathological dimensions, as established by section III of DSM-5. We propose that the PPM may be used as a sanity classification located in the positive and reverse pole of the current psychopathology nosology (Figure 2) that –as well as PID-5′s and BFI dimensions– assesses personality traits from a dimensional point of view (e.g., Leary, 1957; Offer and Sabshin, 1991; Millon and Everly, 1994; Strack and Lorr, 1994; Millon, 1996).

In detail, Serenity is the reverse version of Negative Affect and has a positive component that goes beyond the Emotional Stability (Neuroticism reverse) proposed by the FFM. High scores in this dimension represent subjects characterized by an almost imperturbable peace, who have the capacity to master negative emotions in situations that are out of their control, unpleasant or produce great discomfort. Additionally, these subjects tend to remain calm and maintain an agreeable manner when facing interpersonal conflicts in which their interlocutor experiences great negative emotions. Humanity, on the other hand, is the positive version of Detachment and Extraversion. Higher scores represent individuals that are extremely sensitive to their surroundings and have no difficulties in showing their own vulnerability in the presence of others. These individuals not only have a natural orientation toward others, but they are also compassionate and sympathetic. They are moved by others’ suffering and try to alleviate that suffering. The Integrity dimension emerges as the opposite pole of Antagonism and the positive version of Agreeableness. People with this positive trait are dependable and trustworthy because they are honest about their intentions, keep their promises, tell the truth, and present themselves as they really are. Furthermore, they are humble, have no issues in admitting their mistakes and always expect an egalitarian treatment. The Moderation trait refers to caution and it is placed in the Disinhibition-Conscientiousness continuum. The contents of this trait may seem to not differ greatly from the characteristics assessed by Conscientiousness. However, Pearson correlation between Moderation and Conscientiousness indicated that although they are positively associated, they are not the same construct. An analysis at the content of their items shows that Conscientiousness as measured by the BFI aims at assessing the degree in which someone is organized, reliable, tidy, and fulfills their responsibilities. Moderation, as measure by PTI refers to individuals that characterized that take the necessary time to assess risks and benefits before acting. These individuals plan their actions considering the consequences and tend to avoid unnecessary risks. That is, Moderation assesses if someone tends to take into account the possible consequences of their actions before acting. In contrast, Conscientiousness refers to the occurrence of responsible behavior. We do not know if this was motivated by reflecting on possible consequences or some other reason. Finally, the fifth dimension was labeled as Sprightliness. It includes characteristics of people who know what they want, have clear goals, tend to concentrate their energy on achieving those goals and enjoy what they do every day. Individuals with high scores in this trait feel active, energetic, fulfilled, useful, and confident.

Furthermore, some shared elements between the theory-driven and the data-driven models may be identified when the PPM is compared with previous classifications (Table 3). The PPM includes many aspects of known positive classifications. Serenity, Humanity, and Integrity are the traits that share the most elements with previous models of sanity (Walker and Pitts, 1998; Cawley et al., 2000; Peterson and Seligman, 2004; Leising et al., 2009; Huppert and So, 2013; Morales-Vives et al., 2014; Cosentino and Castro Solano, 2017). In Serenity, elements of self-regulation, self-control, emotional stability, peace, and simply serenity can be outlined. This dimension is related to the postulates of oriental philosophy that underline the importance of serenity as the most important human quality. Buddhists refer to this state of calmness and tranquility as *Passaddhi* –tranquility – or *Samatha* –serenity– (Ñamanoli, 1995).

**Table 3:** Shared element between the PPM and other positive classifications.

	PPM				
	Serenity	Humanity	Integrity	Moderation	Sprightliness
Peterson and Seligman, 2004	Self-regulation	Humanity, Love, Kindness, Clemency and misericord	Integrity, Humility	Prudence	Vitality Zest
Leising et al., 2009	Have self-control	Connect with others emotionally and treat them fairly	Be trusting		
Huppert and So, 2013	Emotional stability				Engagement Vitality
Walker and Pitts, 1998		Kind-hearted	Dependable- Integrity		
Cawley et al., 2000	Serenity	Empathy			
Morales-Vives et al., 2014	Serenity	Compassion	Rectitude		
Cosentino and Castro Solano, 2017	Peace		Honesty		

Regarding Humanity, precedent positive classifications include related elements such as kindness, mercy, empathy, compassion, emotional connection with others, and an agreeable manner. Humanity or compassion emerges as another central aspect in oriental philosophies. Their main theoretical precedent may be situated in Confucius’ concept of *Jen*, which refers to a combination of kindness, humanity, and respect among individuals (Keltner, 2009). The Dalai Lama also highlighted that in the practice of compassion relies the key to happiness (Dalai Lama, 2008). Additionally, from an evolutionary perspective, Darwin (1871) stated that compassion is our strongest instinct. As Kukk (2017) highlighted, Darwin had even given more importance to this concept than to survival of the fittest.

In relation to Integrity, a parallelism may be drawn with elements from previous classifications such as humility, dependability, loyalty, rectitude, honesty, and plain integrity. The HEXACO model –of a psycholexic nature– was proposed as a new version of the FFM in which –similarly to the PPM’s Integrity dimension, an Honesty-Humility factor was included (Ashton and Lee, 2008a). In their analysis, the authors highlighted that, when this sixth factor was added, the model explained more variance of various behaviors than if only the FFM traits were included (Ashton and Lee, 2008b). Here, the Integrity dimension seems to function as a positive trait, in contrast to a neutral trait.

Conversely, Moderation does not have as many precedents as the previous dimensions. Only the prudence strength of the VIA model could be identified (Peterson and Seligman, 2004). However, this dimension has precedents in Greek philosophy. For example, the principle of moderation can be found among Plato and Aristotle’s postulates, which stressed that moderation was an important human quality (Adeel, 2015).

Regarding Sprightliness, Peterson and Seligman’s (2004) and Huppert and So (2013) both outlined the vitality characteristic. In Huppert and So’s (2013) proposal, the engagement notion is also similar to this positive trait. From a theoretical standpoint, the concept of *flow* (Csikszentmihalyi, 1990) may be compared to this dimension since it combines elements of concentration, well-being, and efficacy. However, from a trait point of view, this dimension refers to a trait, stable in time and identifiable in multiple contexts that combine hedony and eudaimony as the flourishing concept defined by Keyes and Haidt (2002). However, in this case, it is conceived as a predisposition, as a way of usual way being, a trait; it is not conceptualized as an outcome.

Finally, the hierarchical multiple regression analysis was intended to determine whether the PPM traits predicted well-being beyond the variance explained by the FFM typical personality traits. This was interpreted as evidence of incremental validity and it was observed, particularly when predicting the total score of mental health as well as its subaspects –emotional, psychological, and social wellbeing–. These results are in line with previous studies which state that positive variables are better predictors of optimal functioning than typical traits (Elston and Boniwell, 2011; Wood et al., 2011; Duan et al., 2012; Gander et al., 2012; Mongrain and Anselmo-Matthews, 2012; Drozd et al., 2014; Meyers and van Woerkom, 2014; Young et al., 2014).

Some of the limitations of this research need to be outlined. Firstly, the sample was non-probabilistic, which may limit the ability to generalize the results to other populations. Additionally, the cross-sectional design limits the interpretation of the data in terms of generalizability since the data here analyzed represent participants in one moment of their lives. As the PPM aims to assess personality traits and personality traits are primarily characterized by their stability over time, a longitudinal study that tests their invariance is mandatory. Longitudinal studies are needed also for testing the PPM predictive capability over different outcomes of interest.

Moreover, the focus of this study was a classical between-person differences analysis. This approach is partial since it lacks the view of the individual processes implied. The need to integrate both individual and inter-individual methodologies of analysis has been emphasized (Borsboom et al., 2003; Beckmann and Wood, 2017). There is evidence that the results obtained by within-subject analyses are different and not captured in between-person approaches. For example, in research that studied and compared both levels of analysis, Beckmann et al. (2010) found reverse results regarding the association between neuroticism and conscientiousness. The variables were negatively correlated in the between-subjects analysis and positively correlated in the within-subject level. This difference may also be applied to the PPM’s positive traits here presented and future research should explore it.

In addition, the originality of the model proposed impacts on its replicability. Although the structure obtained was confirmed in a sample other than the one used in the exploratory analysis, future studies should try to replicate this model in different contexts, cultures, and languages. It should be noted that, just as occurs in mental disorders, the nature of “health” or “positivity” of the traits here introduced might differ from context to context. For example, serenity may be healthy in the long term when common negative emotions or interpersonal conflicts are involved. However, it may be counterproductive in emergency situations or in long-term harmful contexts where individuals may need to defend themselves in a somewhat aggressive way or stop tolerating others’ aggressions. This logic may be applied to all other PPM traits. As Leising (2008) and Leising et al. (2009) pointed out, cultural dependability is an inherent problem to the concept of PDs. Therefore, it would be bold to assume that the PPM has overcome this type of profound psychological dilemmas.

Besides these limitations, we conclude that the five traits of the PPM may be placed in the positive pole of the sickness-health continuum, and we propose it as a possible sanity classification integrated into DSM-5′s dimensional proposal for assessing PDs. Future research should study the predictive ability of the PPM traits on other variables of optimal functioning such as job performance and academic achievement, among others.

## Author Contributions

GdI and ACS contributed substantially to the design and data collection of this research, as well as to the data analysis and interpretation and manuscript writing.

## Conflict of Interest Statement

The authors declare that the research was conducted in the absence of any commercial or financial relationships that could be construed as a potential conflict of interest.
