# Supplementary material for: The Positive Personality Model (PPM): Exploring a New Conceptual Framework for Personality Assessment
Source: Front Psychol. 2018 Oct 25;9:2027. doi: 10.3389/fpsyg.2018.02027 (PMC6209641; doi:10.3389/fpsyg.2018.02027)
Supplement: Supplementary file 1 [file Table_1.pdf]

Supplementary file

Supplementary table 1.
Five factor solution for the PTI-5 (n = 860).

	F1	F2	F3	F4	F5
Item 003	.14	-.05	.07	.07	**.44**
Item 007	.05	.03	.13	-.18	**.63**
Item 008	.02	**.71**	-.03	.03	.01
Item 011	.03	**.69**	.23	-.37	.03
Item 013	.03	**.61**	.13	-.04	-.09
Item 016	.25	-.06	-.01	-.03	**.59**
Item 017	.28	-.05	.02	-.03	**.50**
Item 018	**.51**	-.13	.06	-.04	.17
Item 019	.07	**.61**	.10	.02	-.08
Item 026	.05	-.06	**.71**	.12	-.24
Item 027	.06	.09	**.66**	-.12	-.02
Item 028	**.59**	.11	.15	-.14	.08
Item 029	.01	.06	**.69**	-.29	.24
Item 030	-.03	.16	**.67**	-.12	-.10
Item 032	**.60**	.15	-.04	.05	.01
Item 038	**.63**	.01	-.03	.12	.10
Item 040	.05	.09	.10	**.49**	.02
Item 045	-.10	**.61**	-.23	.34	.04
Item 047	-.16	.02	**.54**	.11	.19
Item 048	-.04	-.10	.03	.15	**.70**
Item 050	.03	-.18	**.85**	-.21	.01
Item 053	-.06	.03	.26	**.42**	.08
Item 056	-.09	-.01	.03	**.62**	.06
Item 061	.05	-.09	**.77**	-.12	-.06
Item 067	-.09	.04	-.07	-.03	**.70**
Item 076	-.04	.14	.08	**.54**	.05
Item 084	-.11	**.71**	-.21	.12	.04
Item 086	.01	-.04	.09	**.73**	.01
Item 088	-.26	.12	**.59**	.14	.09
Item 091	.17	**.66**	.01	-.25	-.01
Item 092	**.65**	.16	.07	-.12	-.11
Item 098	-.05	.13	-.05	-.16	**.73**
Item 102	**.80**	-.11	-.04	-.03	-.02
Item 104	-.05	.07	**.49**	.05	.03
Item 109	**.61**	-.03	.19	-.23	-.07
Item 112	-.12	.01	-.09	.12	**.72**
Item 114	-.08	.05	-.03	**.71**	.05
Item 118	-.06	-.01	**.63**	-.14	.20
Item 124	.07	-.01	**.60**	.06	-.04
Item 130	**.78**	-.17	-.15	.19	-.01
Item 144	-.02	.03	**.58**	-.12	.18
Item 151	.07	.03	**.47**	.26	-.13
Item 156	-.03	.04	.28	**.40**	.03
Item 158	**.75**	-.02	-.03	-.12	-.01
Item 165	**.48**	-.15	.26	-.11	.15
Item 167	-.02	**.68**	.17	-.13	-.03
Item 168	.10	-.04	**.66**	.22	-.22
Item 169	.04	-.01	**.48**	.30	-.16
Item 170	**.53**	.13	-.14	.27	-.03

Item 171	-.06	-.10	.23	**.65**	.06
Item 179	.17	.01	.10	**.51**	-.06
Item 187	.11	.02	.15	**.49**	-.03
Item 188	**.68**	.10	-.13	-.04	.05
Item 199	.05	.12	**.51**	-.17	.20
Item 204	.06	-.11	.05	.19	**.45**
Item 206	.06	-.03	.03	**.68**	.04
Item 207	.14	.18	-.27	**.72**	.02
Item 212	-.04	-.07	**.71**	.21	-.16
Item 214	.09	.09	-.09	**.48**	.15
Item 216	**.50**	.14	-.16	.24	.02
Eigenvalues	16.1	3.94	3.28	2.71	2.22
Cronbach's alphas	.88	.80	.90	.86	.82
Ordinal alphas	.91	.88	.93	.91	.88
Omegas	.89	.85	.92	.87	.84
% Explained variance	12.5	8.8	16.7	12.5	8.9
% of total Explained Variance 59.45%					
Bartlett's Statistic = 20482.0, 1770 *df*, $p < .001$					
Kaiser-Meyer-Olkin (KMO) test = .927					

Supplementary table 2.
Correlations among PTI-5 scales (n = 860).

	Pearson's *r*s (r^2)				
	Flourishing	Integrity	Serenity	Moderation	Humanity
Flourishing	1				
Integrity	.56(.31)**	1			
Serenity	.50(.25)**	.41(.17)**	1		
Moderation	.40(.16)**	.40(.16)**	.47(.22)**	1	
Humanity	.40(.16)**	.48(.23)**	.36(.13)**	.34(.12)**	1

** $p < .01$
